# Mental Health Before and After Retirement—Assessing the Relevance of Psychosocial Working Conditions: The Whitehall II Prospective Study of British Civil Servants

**DOI:** 10.1093/geronb/gbz042

**Published:** 2019-04-11

**Authors:** Maria Fleischmann, Baowen Xue, Jenny Head

**Affiliations:** 1 Department of Health Sciences, Vrije Universiteit Amsterdam, De Boelelaan, The Netherlands; 2 ESRC International Centre for Lifecourse Studies in Society and Health (ICLS), Department of Epidemiology and Public Health, University College London, Gower Street, United Kingdom; 3 Department of Epidemiology and Public Health, University College London, United Kingdom

**Keywords:** General Health Questionnaire, Longitudinal analysis, Occupational cohort study, Work exit, Working environment

## Abstract

**Objectives:**

Retirement could be a stressor or a relief. We stratify according to previous psychosocial working conditions to identify short-term and long-term changes in mental health.

**Method:**

Using data from the Whitehall II study on British civil servants who retired during follow-up (*n* = 4,751), we observe mental health (General Health Questionnaire [GHQ] score) on average 8.2 times per participant, spanning up 37 years. We differentiate short-term (0–3 years) and long-term (4+ years) changes in mental health according to retirement and investigate whether trajectories differ by psychosocial job demands, work social support, decision authority, and skill discretion.

**Results:**

Each year, mental health slightly improved before retirement (−0.070; 95% CI [−0.080, −0.059]; higher values on the GHQ score are indicative of worse mental health), and retirees experienced a steep short-term improvement in mental health after retirement (−0.253; 95% CI [−0.302, −0.205]), but no further significant long-term changes (0.017; 95% CI [−0.001, 0.035]). Changes in mental health were more explicit when retiring from poorer working conditions; this is higher psychosocial job demands, lower decision authority, or lower work social support.

**Discussion:**

Retirement was generally beneficial for health. The association between retirement and mental health was dependent on the context individuals retire from.

Two opposing arguments are made regarding how retirement and mental health are related. Traditionally, retirement was viewed as a stressful transition disrupting individuals’ established routines, with negative consequences for health. However, many authors have challenged this assumption and argued instead that retirement could be viewed as a relief from work, allowing individuals to pursue their own interests and leisure activities. As such, they posited that retirement would contribute positively to mental health. Most studies have shown positive associations between retirement and mental health (Airagnes et al., 2015; Butterworth et al., 2006; Drentea, 2002; Jokela et al., 2010; Mandal & Roe, 2008; Mänty et al., 2018; Mein, Martikainen, Hemingway, Stansfeld, & Marmot, 2003; Nuttman-Shwartz, 2004; Oksanen & Virtanen, 2012; Olesen, Rod, Madsen, Bonde, & Rugulies, 2015; Reitzes, Mutran, & Fernandez, 1996; Westerlund et al., 2010), but some have also found negative (Dave, Rashad, & Spasojevic, 2008; Heller-Sahlgren, 2017; Hyde, Ferrie, Higgs, Mein, & Nazroo, 2004; Wheaton, 1990) or no associations at all (Coe & Zamarro, 2011; Horner & Cullen, 2016; Laaksonen et al., 2012; Mojon-Azzi, Souza-Poza, & Widmer, 2007; Yeung, 2013). Retirement has also been associated with physical health functioning, physical (dis)abilities and (chronic) illnesses (Gall, Evans, & Howard, 1997; Hessel, 2016; Seitsamo & Klockars, 1997; van Zon, Bültmann, Reijneveld, & de Leon, 2016), and self-rated general health (Rijs, Cozijnsen, & Deeg, 2011; Seitsamo & Klockars, 1997; van den Bogaard, Henkens, & Kalmijn, 2016; van Solinge, 2007; Westerlund et al., 2009), with mixed evidence.

In the current social and policy context where working lives are extended and retirement and pension ages increased, it may be of interest to occupational and health care policy advisors to know how mental health develops preceding and following retirement. If individuals encounter adverse health conditions before retirement, this might increase costs for (occupational) health care services, but also for employers due to lower productivity or higher absenteeism of employees. Increases to pension age might prolong this situation. Indeed studies have indicated that adverse health conditions were related to earlier work exit and more absenteeism (e.g., van den Berg, Elders, & Burdorf, 2010). Improvements in health upon retirement could lower the costs for health care providers, but might also present a challenge because individuals would naturally be less inclined to postpone retirement. Work redesign or provision of favorable working conditions could be a possible strategy to improve health before retirement and facilitate later work exit (Ilmarinen, 2006).

In this article, we investigate mental health preceding and following retirement. We contribute to previous research in several ways. First, we assess the association between retirement and mental health longitudinally, making use of unique data spanning more than 30 years. Many previous studies have compared mental health at two time points or compared retirees to workers (Halleröd, Örestig, & Stattin, 2013; Hyde et al., 2004; Mänty et al., 2018; Mein et al., 2003; Mojon-Azzi et al., 2007; Nuttman-Shwartz, 2004; Reitzes et al., 1996; Wheaton, 1990; Yeung, 2013). This can be problematic because retirement is not randomized and selection into retirement might strongly be related to individuals’ health (Oksanen & Virtanen, 2012).

Second, the long follow-up enables us to investigate preretirement changes in mental health. As retirement is an anticipated event (Luhmann, Hofmann, Eid, & Lucas, 2012), mental health could already change preceding retirement; not including this development would bias results.

Third, we investigate both the short-term and the long-term consequences of retirement for mental health. This is based on Atchley’s (1976) retirement adjustment processes hypothesis, stating that retirees experience various phases to adapt to retirement in which mental health could vary. Among more recent studies observing older persons multiple times (i.e., more than twice) in their transitions from work to retirement (Calvo, Sarkisian, & Tamborini, 2013; Dave et al., 2008; Heller-Sahlgren, 2017; Jokela et al., 2010; Mandal & Roe, 2008; Oksanen et al., 2011; Westerlund et al., 2010), only few specifically depict short-term and long-term associations with retirement (Jokela et al., 2010; Oksanen et al., 2011; Westerlund et al., 2010), whereas others tend to present average effects for one or several postretirement periods (Calvo et al., 2013; Dave et al., 2008; Heller-Sahlgren, 2017; Mandal & Roe, 2008).

Finally, we explicitly investigate how health effects of retirement depend on individuals’ working conditions prior to retirement. Rather than generally regarding retirement as either a relief from work or a stressor, we base our expectations on the assumption that retirement could be a relief from an “alienating” and stressful workplace (Drentea, 2002; Olesen et al., 2015; Oshio & Kan, 2017; Stenholm & Vahtera, 2017; van Zon et al., 2016). As such, we consider individuals’ psychosocial working conditions before retirement, that is, psychosocial job demands, decision authority, skill discretion, and work social support, as possibly moderating the association between retirement and mental health. So far, this expectation has frequently been referred to, but to the best of our knowledge no prior study has tested it for mental health. A few studies have, however, investigated similar questions. Wheaton (1990) reports that retirement from a low stress job resulted in an increase of distress symptoms after retirement, whereas retirement from high stress job resulted in a decrease of these symptoms. Moreover, both Westerlund et al. (2009) and Van den Bogaard et al. (2016) showed that a poor working environment before retirement, expressed by low occupational grade, high job demands, and low satisfaction as well as job stress, respectively, was associated with steeper retirement-related improvement in self-rated health.

## Theoretical Considerations

Early research in gerontology viewed retirement as major life event that could produce stress (Ekerdt, 1987) because preestablished routines were disrupted and one’s work or social role lost (van der Heide, van Rijn, Robroek, Burdorf, & Proper, 2013; van Zon et al., 2016). Also in the sociology of work, arguments were put forward that transitions to retirement would have negative effects for mental health (Drentea, 2002), because the empowering conditions accompanying work (money, status, and attachment) were lost. However, reverse arguments were made as well, and most research corroborated a positive association (van der Heide et al., 2013); retirement could be viewed as a health-preserving life change as it relieves individuals from demanding work and provides more time for physical activities (Coe & Zamarro, 2011; Moen, 1996). To this effect, retirement would remove individuals from stressful work situations (Stenholm & Vahtera, 2017).

### Working Conditions

Extending on these notions, it seems virtually impossible to identify the association between retirement and mental health without considering the context in which retirement occurs (Stenholm & Vahtera, 2017; Wheaton, 1990). Individuals work in different environments and whether retirement is experienced as stressful might depend on their previous working conditions (Wheaton, 1990). In situations where older persons retire from stressful working lives, being relieved of these stressors could be beneficial for their health (e.g., Moen, 1996), whereas retirement could induce stress for individuals retiring from a job they enjoy.

Karasek’s job demands–control (support) model relates psychosocial working conditions to stress (Karasek, 1979). It states that working in a high demand and low control job is stressful for individuals and decreases their well-being, whereas combinations of high demand and high control are representative of challenging work and increase well-being. Social support was added as a third dimension to this model, arguing that high support from colleagues and supervisors could help reduce otherwise stressful situations. Workers may experience stress from working in a job with unfavorable psychosocial working conditions. Leaving work for retirement could eliminate such stressors and contribute to individuals’ mental health. In contrast, in circumstances where people work in jobs or environments with favorable psychosocial working conditions, retirement, rather than work, might represent a potential stressor.

Using the exceptionally rich and repeatedly available information on individuals’ mental health and work participation that is available in the Whitehall II occupational cohort study, we aimed to contribute to solving the puzzle of how transitioning to retirement and mental health are associated. In doing so, we explicitly investigate how this relationship varies depending on individuals’ working conditions.

## Methods

We used data from Phases 1–12 of the Whitehall II cohort study, established in 1985–1988 (Phase 1) and described in detail elsewhere (Marmot & Brunner, 2005; Marmot et al., 1991). Phase 10 was a pilot study on a small subsample and therefore not included here. The sample at Phase 1 included 10,308 London-based, male and female civil servants, aged 35–55 years. Two thirds of the respondents were men. Follow-up interviews were conducted every 2–3 years. Response rates varied between 67% and 87%. Data collections alternated between postal questionnaires alone (Phases 2, 4, 6, and 8) and combined postal questionnaires and clinical examination (Phases 1, 3, 5, 7, 9, 11, and 12). Ethical approval for the Whitehall II study was obtained from the University College London Medical School committee on the ethics of human research. As in the general UK population, State Pension age for the Whitehall II cohort was 65 years for men and 60 years for almost all women (increasing to 62 years 8 months for younger women affected by recent changes in State Pension age). Moreover, civil servants have an occupational pension scheme and normal civil service retirement age for this cohort was age 60 years. Early retirement options were available as were options to continue working beyond age 60 years.

### Sample Selection

From the baseline sample (Phase 1) of 10,308, we excluded those who died (*n* = 362) or were censored (*n* =2,730) before leaving work, as well as those who left work before Phase 3 (*n* = 228) or after Phase 11 (*n* = 463), because for these we could not measure mental health before and after retirement. Moreover, we excluded respondents who left work through routes other than retirement (*n* = 1,279). Specifically, we excluded respondents who left work through routes that are not final (unemployment or “other,” e.g., homemaker) and those retiring for health-related reasons. The last decision is based on the possibility that adverse mental health could be the reason, rather than the consequence, of retirement. Finally, we restricted our analyses to those who had repeated measures of mental health, that is, at least one measure before and one after retirement (*n* = 480 excluded). We lost 15 respondents for whom all time points of a covariate or independent variable were missing; therefore, our analytical sample refers to 4,751 unique respondents (38,968 repeated observations).

### Mental Health

We used the 30-item General Health Questionnaire (GHQ) scale to measure mental health (Stansfeld, Head, Fuhrer, Wardle, & Cattell, 2003), which was available in all study Phases 1–12 with the exception of Phase 4. The GHQ scale has been validated for Whitehall II data previously and has good criterion validity for minor psychiatric disorders (Head et al., 2013). The 30 questions, covering depression, anxiety, sleep disturbance, and social functioning, are answered on scales ranging from 0 to 3 (detailed description of the wording are provided in Goldberg (1972)). The GHQ mental health scale ranges from 0 to 100, with higher values indicating worse mental health. Depression cases can be defined as those scoring higher than 5 on the scale (Head et al., 2013), but the scale was used as a continuous indicator here.

### Retirement Age and Time to and Following Retirement

In Phases 3–11, we determined respondents’ employment status and route of work exit by self-report. Participants who reported to be working in the civil service or working outside the civil service were coded as being in work. Participants who were not working during follow-up self-reported the reason for not working. We were interested in those who reported to have “retired” but not particularly “on health grounds.” Retirement is the most frequent transition out of work (81% of those leaving work). As noted earlier, respondents reporting leaving work through other exit routes, that is, unemployment (4%), retirement due to health (9%), or other exit (e.g., homemaker; 5%), were excluded. In case participants reported multiple successive exits from work (10%), the route of work exit refers to their last exit from paid work. Participants who had left the civil service to retire between two data collections reported their year of leaving work. For these (63.7% of the cases), we could calculate their retirement age directly. If participants’ exact retirement age was not available directly, we used the midpoint between their age in the last available phase still in work and the first phase in retirement.

Information on individuals’ retirement age was then used to calculate for each individual and each phase of data collection how long before or after retirement the respective data collection had taken place, that is, the time to retirement and time following retirement. Years before and after retirement were finally rounded and observations 22 years before retirement or earlier and 17 years after retirement or later were omitted from the analyses, due to few observations at those incidences (<1% per year, *n* < 524 per year). Thus, we observe individuals at most 21 years before and 16 years following retirement.

### Psychosocial Working Conditions

All participants who were working in Phases 1–3, 5, and 7 self-reported psychosocial job demands, decision authority, skill discretion, and social support using Karasek’s Job Content Questionnaire (Bosma et al., 1997). Psychosocial job demands were operationalized by four items such as “Do you have to work very fast?” Skill discretion and decision authority are regarded as being indicative of job control. Skill discretion is measured by six items such as “Do you have to do the same thing over and over again” and decision authority by nine items asking among others “Do you have a choice in deciding how to do your work?” Social support at work consists of six items combining aspects of support from colleagues and superiors. For each item, respondents rated whether it is “often,” “sometimes,” “seldom,” or “never/almost never” the case. The final scales for job demands, skill discretion, decision authority, and social support were converted to range from 0 to 100, with higher values indicating higher job demands, skill discretion, etc. For each respondent who had working conditions measured in at least one and at most five phases, we calculated average values based on the available phases.

### Covariates

Analyses were adjusted for sociodemographic variables, health indicators, and health behaviors (see Table 1 for descriptive information) that have previously been related to health outcomes and retirement transitions (Chandola, Ferrie, Sacker, & Marmot, 2007; Hagger-Johnson et al., 2017). Sociodemographic variables were sex (female = 1, male = 0), year of birth, age at retirement, and partnership status (married/cohabiting; single; divorced; widowed). Partnership status was assessed in each study phase (missing observations were filled with information from preceding phases) and is included as time-varying indicator. Occupational grade level was a time-varying indicator measured in Phases 1–11 distinguishing three categories (high = administrative; middle = professional/executive; low = clerical/support). If individuals took on work outside the civil service or retired, their occupational grade level is no longer reported, but we used information from preceding phases. In addition, we included an indicator whether people were still working in the civil service at retirement. Moreover, we accounted for health measures and health behaviors, all of which are time-varying: intake of medicine due to depression (yes/no, observed in Phases 4–12, and missing phases and observations were imputed with information from later phases), diagnose of chronic illness (yes/no indicator of validated measures of diabetes, coronary heart disease [excluding self-report], all stroke, and all malignant cancers, observed in Phases 1–9, and later phases were imputed with information given previously), smoking status (current smoker; ex-smoker; never smoker; observed in Phases 1–3, 5, 7, 9–12, and missing phases and observations were filled in with information from previous phase), alcohol dependence (yes/no; observed in Phases 3, 5, 7–11, and missing phases and observations were filled with information from following or previous phase), and body mass index (BMI in kg/m^2^; observed in odd-numbered phases and Phase 12 and missing phases and observations were imputed with information from previous phase) in categories (<25 normal or underweight; 25–30 overweight; 30+ obese). Item nonresponse is relatively low for the variables described earlier (<5%), with few exceptions (e.g., for BMI) and is described in the Supplementary Table 1.

**Table 1.** Descriptive Information for Analysis Sample (Observations = 38,968)

	Mean (SD)	%
Mental health (GHQ score)	2.68 (4.99)	
Psychosocial working conditions		
Psychosocial job demands	60.49 (15.34)	
Work social support	75.99 (13.68)	
Skill discretion	71.15 (15.51)	
Decision authority	65.62 (14.09)	
Women		29.33
Year of birth	1941 (5.88)	
Age left work	60.54 (4.46)	
Partnership status		
Married/cohabiting (ref.)		76.66
Single		12.20
Divorced		6.87
Widowed		4.27
Occupational grade level		
Administrative (ref.)		44.70
Professional/executive		43.39
Clerical/support		11.91
Left civil service		32.80
Depression medication intake (no)		97.51
Chronic illness (no)		82.84
Smoking status		
Never smoker (ref.)		49.63
Ex-smoker		41.62
Current smoker		8.74
Alcohol dependency (no)		90.60
Body Mass Index (BMI)		
Normal or underweight (BMI <25; ref.)		45.87
Overweight (BMI 25–30)		40.46
Obese (BMI 30+)		13.67

### Statistical Analyses

Respondents were observed at least twice and at most 10 times, and on average contributed 8.2 observations, implying that time is nested within participants. We conducted multilevel random effects linear regression analyses using *xtmixed* in Stata 15.0.

**Figure 1.**
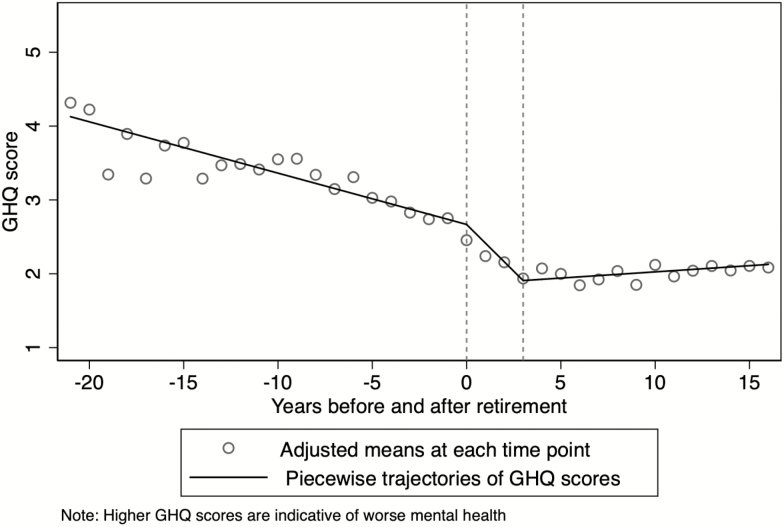
Development of mental health (General Health Questionnaire [GHQ] score) dependent on retirement.

To investigate mental health before and after retirement and to accommodate ideas about retirement adjustment processes, we defined three time-related splines (i.e., piecewise trajectories), one for the period preceding retirement (i.e., −21 to −1 years before retirement), one identifying short-term effects of retirement (i.e., 0–3 years after retirement), and one covering the long-term effects (i.e., 4–16 years after retirement). In Figure 1, we show, by depicting the piecewise trajectories in combination with the adjusted means for each time point, that the three splines are a good approximation of our data. To identify whether the association between retirement and mental health is moderated by the four psychosocial working conditions, we include interactions between the three splines and each measure of working conditions in the analyses, while holding constant for the remaining three working conditions.

To check the robustness of our results, we implemented additional analyses. First, we included an additional spline covering the 3 years preceding retirement. We did this, because retirement is a predictable work transition and effects on mental health could precede the actual transition from work into retirement (Luhmann et al., 2012). Second, we restricted our sample to participants for whom retirement age could be calculated directly. As explained earlier, this was the case for 63.4% of the sample (*n* = 3,032). This helps eliminating possible bias induced by imprecise identification of retirement age and identifying a more precise measure of the effect magnitude of retirement for mental health.

We estimated combined models for women and men, as additional analyses did not reveal significant gender differences in the association between retirement and mental health (results available from authors upon request).

## Results

We depict descriptive statistics of our sample in Table 1. Of all participants, 70% were men and the mean age at which they left work was 60.5 years. More than three quarters were married or cohabiting. Of all participants, 45% worked in the highest occupational grade (administrative), and another 43% in the middle occupational grade (professional/executive). Upon retirement, 33% had left civil service. Regarding health measures and health behaviors, nearly 98% did not take depression medication and 83% had no chronic illness. Nearly half of the participants were never smokers and more than 90% categorized without alcohol dependence. Of the participants, 46% had a BMI less than 25 (normal or underweight) and about 41% had a BMI of 25–30 (overweight). The mean mental health (GHQ) score was 2.68, and psychosocial working conditions were on average between 60 and 76 on scales from 0 to 100.

We present all multivariate results graphically. In addition, Table 2 depicts the estimated regression coefficients for all variables included in the models and Table 3 the results from the moderations. Figure 1 (based on Table 2) shows adjusted mean scores for mental health for each year before and after retirement in combination with the piecewise trajectories. We clearly see that mental health (GHQ scores) changes dependent on retirement. Note that lower GHQ scores represent better mental health. Specifically, individuals’ mental health slightly improves each year preceding retirement (i.e., GHQ scores decline; β = −0.070; 95% CI [−0.080, −0.059]). Upon retirement and up to 3 years after retirement, individuals experience a steep improvement of mental health per year (β = −0.253; 95% CI [−0.302, −0.205]), whereas in the years thereafter mental health slightly, but nonsignificantly, declines each year (β = 0.017; 95% CI [−0.001, 0.035]).

**Table 2.** Multilevel Random Effects Linear Regression Analysis for Association of Retirement (Defined by Three Splines) and Mental Health

	Model 1: Basic Coef. [95% CI]
Change per year (slope)	
Before retirement (−21; −1 y)	−0.070^***^ [−0.080, −0.059]
After retirement short term (0;3 y)	−0.253^***^ [−0.302, −0.205]
After retirement long term (4+ y)	0.017 [−0.001, 0.035]
Psychosocial job demands	0.044^***^ [0.037, 0.050]
Work social support	−0.037^***^ [−0.044, −0.031]
Skill discretion	−0.032^***^ [−0.040, −0.024]
Decision authority	−0.007 [−0.014, 0.001]
Women	0.727^***^ [0.520, 0.935]
Partnership status (ref.: married/cohabiting)	
Single	0.109 [−0.134, 0.352]
Divorced	0.663^***^ [0.410, 0.915]
Widowed	1.068^***^ [0.786, 1.351]
Year of birth	0.003 [−0.013, 0.019]
Occupational grade level (ref.: Administrative)	
Professional/Executive	0.070 [−0.102, 0.242]
Clerical/support	−0.273 [−0.568, 0.022]
Left civil service	0.175 [−0.044, 0.394]
Depression medication intake (no)	−1.685^***^ [−2.017, −1.354]
Chronic illness (no)	−0.377^***^ [−0.540, −0.215]
Smoking status (ref.: never)	
Ex-smoker	−0.088 [−0.255, 0.079]
Current smoker	0.161 [−0.086, 0.408]
Alcohol dependency (no)	−0.876^***^ [−1.074, −0.678]
Body mass index (ref.: Normal or underweight; BMI <25)	
Overweight (BMI 25–30)	−0.085 [−0.213, 0.044]
Obese (BMI 30+)	0.002 [−0.201, 0.204]
Age left work	−0.018 [−0.042,0.007]
Constant	4.470 [−26.893,35.832]
*N* (observations)	38,968
*N* (participants)	4,751
AIC^a^	225916.9
BIC^b^	226156.8

^a^Akaike Information Criterion (AIC). bBayesian Information Criterion (BIC).

*p < .05. **p < .01. ***p < .001.

**Table 3.** Multilevel Random Effects Linear Regression Analysis for Association of Retirement (Defined by Three Splines) and Mental health, Moderated by Psychosocial Working Conditions. Only Moderations Are Shown. Models Are Adjusted for All Variables From Table 2

	Model 2: Psychosocial job demands	Model 3: Work social support	Model 4: Skill discretion	Model 5: Decision authority
	Coef. [95% CI]	Coef. [95% CI]	Coef. [95% CI]	Coef. [95% CI]
Moderation with psychosocial working condition				
Before retirement (−21; −1 y)	−0.002^***^ [−0.002, −0.001]	0.000 [−0.000,0.001]	−0.001* [−0.001, −0.000]	−0.001 [−0.001, 0.000]
After retirement short term (0;3 y)	−0.005^***^ [−0.009, −0.002]	0.008^***^ [0.005, 0.012]	0.001 [−0.002, 0.004]	0.005^**^ [0.002, 0.009]
After retirement long term (4+ y)	−0.001 [−0.002, 0.000]	0.000 [−0.001, 0.002]	0.000 [−0.001, 0.001]	−0.001 [−0.002, 0.001]
*N* (observations)	38,968	38,968	38,968	38,968
*N* (participants)	4,751	4,751	4,751	4,751
AIC^a^	225825.4	225863.9	225918.1	225913.1
BIC^b^	226091.1	226129.6	226183.8	226178.8

^a^Akaike Information Criterion (AIC). bBayesian Information Criterion (BIC).

*p < .05. **p < .01. ***p < .001.

### Working Conditions

**Figure 2.**
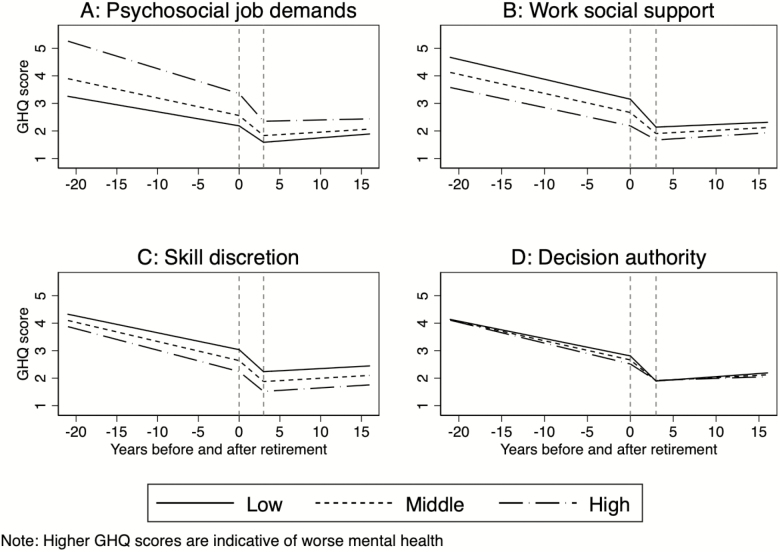
Development of mental health (General Health Questionnaire [GHQ] score) dependent on retirement, moderated by psychosocial working conditions.

We analyzed whether the association between retirement and mental health was moderated by context, that is, individuals’ previous psychosocial working conditions (see Table 3). Working conditions were continuous variables, but for better interpretability we show three groups of workers in the graphs (see Figure 2); that is, those with high (at 75% cutoff), middle (50% cutoff = modal), and low (at 25% cutoff) values on the respective indicator of working conditions. Note that high decision authority, skill discretion, and work social support are categorized as favorable, whereas high psychosocial job demands are unfavorable working conditions. The fundamental association of retirement and mental health is similar to the one described earlier, but several differences are visible according to prior psychosocial working conditions. Most generally, those with favorable working conditions report better mental health before retirement, compared to those with poorer working conditions. Upon retirement, mental health improves for all groups, but improvements are more pronounced for those from poor working conditions. In more detail, we see the following.

First, psychosocial job demands significantly moderate the association between retirement and mental health. Prior to retirement, those reporting the highest psychosocial job demands have highest values of mental health, indicating worst mental health (Figure 2A). Already in the years preceding retirement, workers with higher job demands experience a significantly larger improvement in mental health (β = −0.002; 95% CI [−0.002, −0.001]), and also in the first 3 years following retirement, this improvement is more pronounced (β = −0.005; 95% CI [−0.009, −0.002]), resulting in a reduction in differences in mental health. In the long term after retirement, job demands do not moderate changes in mental health (β = −0.001; 95% CI [−0.002, 0.000]). Second, workers with lower work social support prior to retirement report worse mental health (Figure 2B). Before retirement, the improvement in mental health proceeds independently from work social support (β = 0.000; 95% [CI −0.000, 0.001]). In the 3 years after retirement, the improvement in mental health is largest for those with low social support (β = 0.008; 95% CI [0.005, 0.012]) and flatter with increasing work social support. In the long term after retirement, no additional convergence is noted (β = 0.000; 95% CI [−0.001, 0.002]). Third, the association between retirement and mental health is largely independent of levels of skill discretion (Figure 2C). Only in the years preceding retirement do we find some a minor amplification of the differences in mental health (β = −0.001; 95% CI [−0.001, −0.000]). Finally, individuals with different levels of decision authority report statistically similar mental health both before retirement and in the long term after retirement (Figure 2D). However, in the first years following retirement, the improvement in mental health per year is more pronounced for those with lower decision authority and flatter with higher decision authority (β = 0.005; 95% CI [0.002, 0.009]). Summarizing these results, we find that participants leaving work from a more disadvantageous working environment experience more substantial improvements in mental health in the short term after retirement; these improvements are maintained in the long run, significantly reducing differences between workers from poor and favorable working conditions.

### Sensitivity Analyses

We added an additional spline for the 3 years preceding retirement to accommodate potential anticipatory effects of retirement on mental health. Results (Supplementary Figure 1) are comparable to the ones described earlier. Mental health slightly but significantly improves when approaching retirement. AIC (225912.3) and BIC (226160.8) scores do not substantially improve (full results available upon request) compared to the simpler model with three splines as reported in Table 2 (AIC: 225916.9; BIC: 226156.8).

Restricting the sample to participants whose retirement age was calculated directly (Supplementary Figure 2), we generally find what is presented in Figure 1. The only remarkable difference is that the improvement in mental health in the short term after retirement is more pronounced (slope = −0.369; 95% CI [−0.429, −0.309]), compared to the general model (Table 2; slope= −0.253; 95% CI [−0.302, −0.205]). The steeper slope might result of the more precise measurement of the exact timing of leaving work.

## Discussion

Relating to the persistent question of how retirement and mental health are related, we set out to investigate the short-term and long-term consequences of retirement for individuals’ mental health. Following previous literature emphasizing to consider the work context before retirement, we explicitly considered previous working conditions as moderators. We made use of extensive longitudinal information from the Whitehall II prospective occupational cohort study on British Civil Servants, following individuals for up to 37 years.

Our results showed that retirement was generally related to improvements in mental health. Especially in the 3 years following retirement, individuals on average experienced steep improvements in mental health, and maintained these improvements in the long term following retirement. This finding in principle agrees with the pattern Atchley (1976) described in his retirement adjustment hypothesis: After an initial honeymoon stage, individuals settle into a stability phase. Moreover, our results are in line with previous research showing positive associations between retirement and mental health, and support the theoretical argument that retirement can be a relief and contribute positively to health (e.g., Moen, 1996). We identified one previous publication looking specifically at short-term and long-term effects of retirement on mental health (Heller-Sahlgren, 2017). The authors followed individuals’ mental health, measured by the Euro-D scale, at most 8 years after retirement. Their results oppose our findings: Heller-Sahlgren (2017) identified no short-term effects of retirement for depressive tendencies, but showed increases in the long term. In contrast to our study, focusing on a British sample only, they included individuals from 10 different European countries in their analyses. Whether or not individuals experience retirement as stressful or relieving might be dependent on previous work experiences or institutional factors, such as retirement policies and provision and generosity of pension benefits. This variation in possible retirement experiences between countries is not accounted for in Heller-Sahlgren’s publication and might explain contradicting results. Similar to other studies, they made use of an instrumental variables approach, but other studies using this approach have reported positive health effects of retirement (Hessel, 2016), rather than negative.

With regard to psychosocial working conditions, we showed that individuals retiring from poorer working conditions experienced more pronounced improvements in mental health upon retirement. This was evident for psychosocial job demands, work social support, and decision authority. This finding supports theoretical ideas that the context individuals retire from partly affects their experiences in retirement (Wheaton, 1990). Retiring from a stressful or “alienating” job was argued to be a greater relief for individuals (Drentea, 2002; Olesen et al., 2015; Oshio & Kan, 2017; Stenholm & Vahtera, 2017; van Zon et al., 2016). Previous studies have reported similar associations: Retirement was found to decrease distress symptoms specifically among workers from high stress jobs (Wheaton, 1990), and self-rated health improved more when retiring from a poor working environment (van den Bogaard et al., 2016; Westerlund et al., 2009). For skill discretion, we found that those with lower levels had on average worse mental health, but we did not find, like for the other working conditions, that individuals retiring from jobs with worse conditions, that is, lower skill discretion, had more pronounced improvements in their mental health. This might suggest that leaving a job with lower skill discretion is less of a “relief” for individuals, and therefore, did not positively affect their mental health upon retirement. Relatedly, other studies reported weak associations for skill discretion and minor psychiatric morbidity (Stansfeld, Fuhrer, Shipley, & Marmot, 1999) and depressive symptoms (Theorell et al., 2015), potentially suggesting that in comparison to other working conditions, skill discretion is perceived less of a stressor.

### Contributions and Limitations

We aimed at contributing to the ongoing debate whether retirement and mental health are negatively or positively associated. Unique data, covering up to 21 years preceding retirement and 16 years following retirement, allowed us to observe mental health repeatedly before retirement, as well as specifically address both short-term and long-term changes in mental health upon retirement. We were able to measure mental health at least twice and on average 8.2 times per person. To the best of our knowledge, this is the first study that has such extensive information on mental health both before and after retirement. We show that mental health already slightly improves before retirement; one possible explanation might be the positive outlook for the future (Lachman, Teshale, & Agrigoroaei, 2015), or so-called anticipation effects of retirement (Luhmann et al., 2012). Moreover, the long period of follow-up allowed us to assess slopes of mental health and compare changes in slopes as individuals retire. This is advantageous over previous studies comparing mental health at two time points, before and after retirement, or across groups, those retiring versus remaining in work. The long follow-up of the same group of older persons could also lower the risk of reverse causality.

Despite these advantages, our study has some potential limitations. We could not fully eliminate the possibility of reverse causation. Some people were followed longer than others and the loss to follow-up could bias our results, especially toward the beginning and end of the observation period. However, we dropped very distant observations, that is, those more than 21 years preceding or 16 years following retirement. Our measurement of when exactly retirement occurred for individuals could be slightly distorted because we did not have information on the exact year of retirement for all participants. However, our sensitivity analyses confirmed that mental health improved slightly before retirement, and particularly in the short term following retirement. The Whitehall II sample on civil servants in London is an occupational cohort study. Even though it is selective, it generally is a good representation of the potential variability in the population with white-collar jobs. Moreover, our results were consistent with other studies investigating working conditions as potential moderators from the Netherlands (van den Bogaard et al., 2016) and France on general health (Westerlund et al., 2009) and the United States for mental health (Wheaton, 1990).

## Conclusion

We find that mental health improves following retirement, especially for those coming from more disadvantageous working conditions, and that retirees report an improvement in mental health especially shortly after retirement. This study once again confirms that workers in “good jobs” have better outcomes with regard their mental health, an advantage that those stemming from worse working environments cannot obtain, even though their improvements upon retirement are steeper. In order to potentially reduce health care costs, occupational interventions, offering workers good working conditions early on, might be a solution.

## Supplementary Material

gbz042_suppl_Supplementary_Material

## References

[CIT0001] Airagnes G., Lemogne C., Consoli S. M., Schuster J. P., Zins M., & Limosin F (2015). Personality moderates the improvement of depressive symptoms after retirement: Evidence from the GAZEL cohort. The American Journal of Geriatric Psychiatry, 23, 941–949. doi:10.1016/j.jagp.2014.12.004 25577304

[CIT0002] Atchley R. C (1976). The sociology of retirement. New York: John Wiley & Sons Ltd.

[CIT0003] van den Berg T. I. J., Elders L. A. M., & Burdorf A (2010). Influence of health and work on early retirement. Journal of Occupational and Environmental Medicine, 52, 576–583. doi:10.1097/JOM.0b013e3181de8133 20523241

[CIT0004] van den Bogaard L., Henkens K., & Kalmijn M (2016). Retirement as a relief? The role of physical job demands and psychological job stress for effects of retirement on self-rated health. European Sociological Review, 32, 295–306. doi:10.1093/esr/jcv135

[CIT0005] Bosma H., Marmot M. G., Hemingway H., Nicholson A. C., Brunner E., & Stansfeld S. A (1997). Low job control and risk of coronary heart disease in Whitehall II (prospective cohort) study. BMJ, 314, 558–565. doi:10.1136/bmj.314.7080.558 9055714 PMC2126031

[CIT0006] Butterworth P., Gill S. C., Rodgers B., Anstey K. J., Villamil E., & Melzer D (2006). Retirement and mental health: Analysis of the Australian national survey of mental health and well-being. Social Science & Medicine (1982), 62, 1179–1191. doi:10.1016/j.socscimed.2005.07.013 16171915

[CIT0007] Calvo E., Sarkisian N., & Tamborini C. R (2013). Causal effects of retirement timing on subjective physical and emotional health. The Journals of Gerontology. Series B, Psychological Sciences and Social sciences, 68, 73–84. doi:10.1093/geronb/gbs097 23149431

[CIT0008] Chandola T., Ferrie J., Sacker A., & Marmot M (2007). Social inequalities in self reported health in early old age: Follow-up of prospective cohort study. BMJ, 334, 990. doi:10.1136/bmj.39167.439792.55 17468119 PMC1867907

[CIT0009] Coe N. B., & Zamarro G (2011). Retirement effects on health in Europe. Journal of Health Economics, 30, 77–86. doi:10.1016/j.jhealeco.2010.11.002 21183235 PMC3972912

[CIT0010] Dave D., Rashad I., & Spasojevic J (2008). The effects of retirement on physical and mental health outcomes. Southern Economic Journal, 75, 497–523. doi:10.3386/w12123

[CIT0011] Drentea P (2002). Retirement and mental health. Journal of Aging and Health, 14, 167–194. doi:10.1177/089826430201400201 11995739

[CIT0012] Ekerdt D. J (1987). Why the notion persists that retirement harms health. The Gerontologist, 27, 454–457. doi:10.1093/geront/27.4.454 3623143

[CIT0013] Gall T. L., Evans D. R., & Howard J (1997). The retirement adjustment process: Changes in the well-being of male retirees across time. The Journals of Gerontology. Series B, Psychological Sciences and Social sciences, 52, P110–P117. doi:10.1093/geronb/52B.3.P110 9158562

[CIT0014] Goldberg D. P (1972). The detection of psychiatric illness by questionnaire. London: Oxford University Press.

[CIT0015] Hagger-Johnson G., Carr E., Murray E., Stansfeld S., Shelton N., Stafford M., & Head J (2017). Association between midlife health behaviours and transitions out of employment from midlife to early old age: Whitehall II cohort study. BMC Public Health, 17, 82. doi:10.1186/s12889-016-3970-4 28095887 PMC5240357

[CIT0016] Halleröd B., Örestig J., & Stattin M (2013). Leaving the labour market: The impact of exit routes from employment to retirement on health and wellbeing in old age. European Journal of Ageing, 10, 25–35. doi:10.1007/s10433-012-0250-8 28804280 PMC5549229

[CIT0017] Head J., Stansfeld S. A., Ebmeier K. P., Geddes J. R., Allan C. L., Lewis G., & Kivimäki M (2013). Use of self-administered instruments to assess psychiatric disorders in older people: validity of the General Health Questionnaire, the Center for Epidemiologic Studies Depression Scale and the self-completion version of the revised Clinical Interview Schedule. Psychological Medicine, 43, 2649–2656. doi:10.1017/S0033291713000342 23507136 PMC3821376

[CIT0018] van der Heide I., van Rijn R. M., Robroek S. J., Burdorf A., & Proper K. I (2013). Is retirement good for your health? A systematic review of longitudinal studies. BMC Public Health, 13, 1180. doi:10.1186/1471-2458-13-1180 24330730 PMC4029767

[CIT0019] Heller-Sahlgren G (2017). Retirement blues. Journal of Health Economics, 54(Suppl. C), 66–78. doi:10.1016/j.jhealeco.2017.03.007 28505541

[CIT0020] Hessel P (2016). Does retirement (really) lead to worse health among European men and women across all educational levels? Social Science & Medicine (1982), 151, 19–26. doi:10.1016/j.socscimed.2015.12.018 26773290

[CIT0021] Horner E. M., & Cullen M. R (2016). The impact of retirement on health: Quasi-experimental methods using administrative data. BMC Health Services Research, 16, 68. doi:10.1186/s12913-016-1318-5 26891722 PMC4759763

[CIT0022] Hyde M., Ferrie J., Higgs P., Mein G., & Nazroo J (2004). The effects of pre-retirement factors and retirement route on circumstances in retirement: Findings from the Whitehall II study. Ageing and Society, 24, 279–296. doi:10.1017/S0144686X03001624

[CIT0023] Ilmarinen J (2006). The ageing workforce–challenges for occupational health. Occupational medicine (Oxford, England), 56, 362–364. doi:10.1093/occmed/kql046 16931565

[CIT0024] Jokela M., Ferrie J. E., Gimeno D., Chandola T., Shipley M. J., Head J., … Kivimäki M (2010). From midlife to early old age: Health trajectories associated with retirement. Epidemiology (Cambridge, Mass.), 21, 284–290. doi:10.1097/EDE.0b013e3181d61f53 PMC3204317 20220519

[CIT0025] Karasek R. A (1979). Job demands, job decision latitude, and mental strain: Implications for job redesign. Administrative Science Quarterly, 24, 285–308. doi:10.2307/2392498

[CIT0026] Laaksonen M., Metsä-Simola N., Martikainen P., Pietiläinen O., Rahkonen O., Gould R., … Lahelma E (2012). Trajectories of mental health before and after old-age and disability retirement: A register-based study on purchases of psychotropic drugs. Scandinavian Journal of Work, Environment & Health, 38, 409–417. doi:10.5271/sjweh.3290 22411588

[CIT0027] Lachman M. E., Teshale S., & Agrigoroaei S (2015). Midlife as a pivotal period in the life course: Balancing growth and decline at the crossroads of youth and old age. International Journal of Behavioral Development, 39, 20–31. doi:10.1177/0165025414533223 25580043 PMC4286887

[CIT0028] Luhmann M., Hofmann W., Eid M., & Lucas R. E (2012). Subjective well-being and adaptation to life events: A meta-analysis. Journal of Personality and Social Psychology, 102, 592–615. doi:10.1037/a0025948 22059843 PMC3289759

[CIT0029] Mandal B., & Roe B (2008). Job loss, retirement and the mental health of older Americans. The Journal of Mental Health Policy and Economics, 11, 167–176. doi:10.2139/ssrn.991134 19096091

[CIT0030] Mänty M., Kouvonen A., Lallukka T., Lahti J., Lahelma E., & Rahkonen O (2018). Changes in physical and mental health functioning during retirement transition: A register-linkage follow-up study. European Journal of Public Health, 28, 805–809. doi:10.1093/eurpub/cky013 29425301

[CIT0031] Marmot M., & Brunner E (2005). Cohort Profile: The Whitehall II study. International Journal of Epidemiology, 34, 251–256. doi:10.1093/ije/dyh372 15576467

[CIT0032] Marmot M. G., Smith G. D., Stansfeld S., Patel C., North F., Head J., … Feeney A (1991). Health inequalities among British civil servants: The Whitehall II study. Lancet (London, England), 337, 1387–1393. doi:10.1016/0140-6736(91)93068-K 1674771

[CIT0033] Mein G., Martikainen P., Hemingway H., Stansfeld S., & Marmot M (2003). Is retirement good or bad for mental and physical health functioning? Whitehall II longitudinal study of civil servants. Journal of Epidemiology and Community Health, 57, 46–49. doi:10.1136/jech.57.1.46 12490648 PMC1732267

[CIT0034] Moen P (1996). A life course perspective on retirement, gender, and well-being. Journal of Occupational Health Psychology. 1, 131–144. doi:10.1037/1076-8998.1.2.131 9547042

[CIT0035] Mojon-Azzi S., Sousa-Poza A., & Widmer R (2007). The effect of retirement on health: A panel analysis using data from the Swiss Household Panel. Swiss Medical Weekly, 137, 581–585. doi:2007/41/smw-11841 17990151 10.4414/smw.2007.11841

[CIT0036] Nuttman-Shwartz O (2004). Like a high wave: Adjustment to retirement. The Gerontologist, 44, 229–236. doi:10.1093/geront/44.2.229 15075419

[CIT0037] Oksanen T., Vahtera J., Westerlund H., Pentti J., Sjösten N., Virtanen M., … Kivimäki M (2011). Is retirement beneficial for mental health?: Antidepressant use before and after retirement. Epidemiology (Cambridge, Mass.), 22, 553–559. doi:10.1097/EDE.0b013e31821c41bd PMC3132597 21502864

[CIT0038] Oksanen T., & Virtanen M (2012). Health and retirement: A complex relationship. European Journal of Ageing, 9, 221–225. doi:10.1007/s10433-012-0243-7 28804421 PMC5549195

[CIT0039] Olesen K., Rod N. H., Madsen I. E., Bonde J. P., & Rugulies R (2015). Does retirement reduce the risk of mental disorders? A national registry-linkage study of treatment for mental disorders before and after retirement of 245,082 Danish residents. Occupational and Environmental Medicine, 72, 366–372. doi:10.1136/oemed-2014-102228 25814269 PMC4413684

[CIT0040] Oshio T., & Kan M (2017). The dynamic impact of retirement on health: Evidence from a nationwide ten-year panel survey in Japan. Preventive Medicine, 100(Suppl. C), 287–293. doi:10.1016/j.ypmed.2017.04.007 28583660

[CIT0041] Reitzes D. C., Mutran E. J., & Fernandez M. E (1996). Does retirement hurt well-being? Factors influencing self-esteem and depression among retirees and workers. The Gerontologist, 36, 649–656. doi:10.1093/geront/36.5.649 8942108

[CIT0042] Rijs K. J., Cozijnsen R., & Deeg D. J. H (2011). The effect of retirement and age at retirement on self-perceived health after three years of follow-up in Dutch 55–64-year-olds. Ageing and Society, 32, 281–306. doi:10.1017/S0144686X11000237

[CIT0043] Seitsamo J., & Klockars M (1997). Aging and changes in health. Scandinavian Journal of Work, Environment & Health, 23, 27–35. Retrieved from http://www.jstor.org/stable/40966689 9247993

[CIT0044] van Solinge H (2007). Health change in retirement: A longitudinal study among older workers in the Netherlands. Research on Aging, 29, 225–256. doi:10.1177/0164027506298223

[CIT0045] Stansfeld S. A., Fuhrer R., Shipley M. J., & Marmot M. G (1999). Work characteristics predict psychiatric disorder: prospective results from the Whitehall II Study. Occupational and Environmental Medicine, 56, 302–307. Retrieved from https://www.ncbi.nlm.nih.gov/pubmed/10472303 10472303 10.1136/oem.56.5.302 PMC1757742

[CIT0046] Stansfeld S. A., Head J., Fuhrer R., Wardle J., & Cattell V (2003). Social inequalities in depressive symptoms and physical functioning in the Whitehall II study: Exploring a common cause explanation. Journal of Epidemiology and Community Health, 57, 361–367. doi:10.1136/jech.57.5.361 12700221 PMC1732450

[CIT0047] Stenholm S., & Vahtera J (2017). Does retirement benefit health? Preventive Medicine, 100(Suppl. C), 294–295. doi:10.1016/j.ypmed.2017.05.007 28583661

[CIT0048] Theorell T., Hammarström A., Aronsson G., Träskman Bendz L., Grape T., Hogstedt C., … Hall C (2015). A systematic review including meta-analysis of work environment and depressive symptoms. BMC Public Health, 15, 738. doi:10.1186/s12889-015-1954-4 26232123 PMC4522058

[CIT0049] Westerlund H., Kivimäki M., Singh-Manoux A., Melchior M., Ferrie J. E., Pentti J., … Vahtera J (2009). Self-rated health before and after retirement in France (GAZEL): A cohort study. Lancet (London, England), 374, 1889–1896. doi:10.1016/S0140-6736(09)61570-1 19897238

[CIT0050] Westerlund H., Vahtera J., Ferrie J. E., Singh-Manoux A., Pentti J., Melchior M., … Kivimäki M (2010). Effect of retirement on major chronic conditions and fatigue: French GAZEL occupational cohort study. BMJ, 341, c6149. doi:10.1136/bmj.c6149 21098617 PMC2990862

[CIT0051] Wheaton B (1990). Life transitions, role histories, and mental health. American Sociological Review, 55, 209–223. doi:10.2307/2095627

[CIT0052] Yeung D. Y (2013). Is pre-retirement planning always good? An exploratory study of retirement adjustment among Hong Kong Chinese retirees. Aging & Mental Health, 17, 386–393. doi:10.1080/13607863.2012.732036 23072256

[CIT0053] van Zon S. K. R., Bültmann U., Reijneveld S. A., & de Leon C. F. M (2016). Functional health decline before and after retirement: A longitudinal analysis of the Health and Retirement Study. Social Science & Medicine, 170(Suppl. C), 26–34. doi:10.1016/j.socscimed.2016.10.002 27741444

