# Supplementary material for: Mental Health Before and After Retirement—Assessing the Relevance of Psychosocial Working Conditions: The Whitehall II Prospective Study of British Civil Servants
Source: J Gerontol B Psychol Sci Soc Sci. 2019 Apr 11;75(2):403–13. doi: 10.1093/geronb/gbz042 (PMC7392102; doi:10.1093/geronb/gbz042)
Supplement: gbz042_suppl_Supplementary_Material [file gbz042_suppl_supplementary_material.docx]

Supplementary material


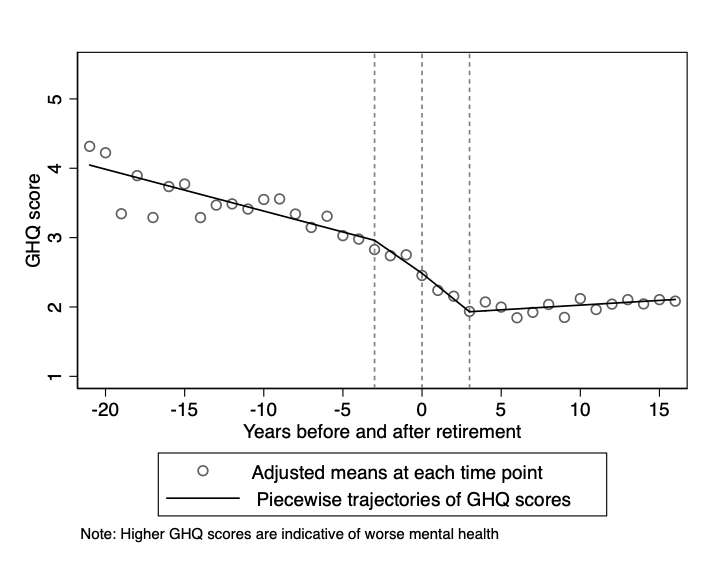


Supplementary Figure 1. Development of mental health (GHQ score) dependent on retirement, including an additional spline preceding retirement.


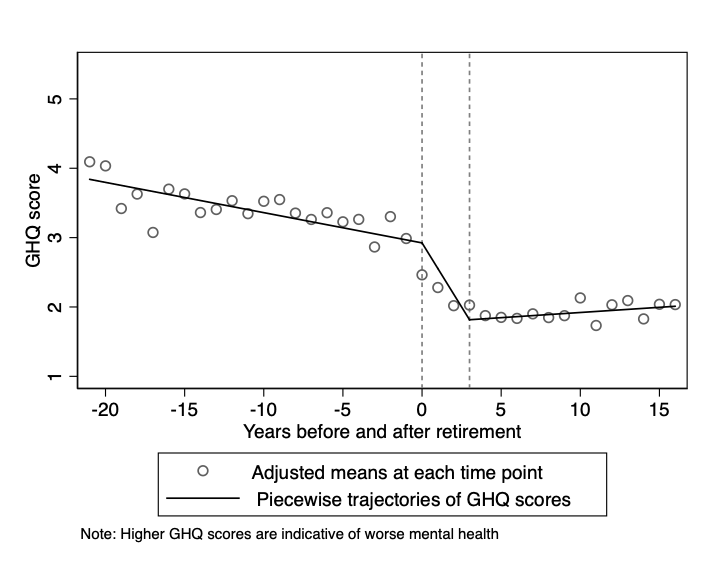


Supplementary Figure 2. Development of mental health (GHQ score) dependent on retirement for participants whose retirement age could be calculated directly (63.4% of sample).

Supplementary Table 1. Item non-response for each variable included in the analyses, by study phase.

|  | Phase 1 | Phase 2 | Phase 3 | Phase 5 | Phase 6 | Phase 7 | Phase 8 | Phase 9 | Phase 11 | Phase 12 |
| --- | --- | --- | --- | --- | --- | --- | --- | --- | --- | --- |
| Observations^a^: | 3,903 | 4,156 | 4,508 | 4,233 | 4,056 | 4,221 | 4,276 | 4,006 | 3,152 | 2,457 |
| Psychosocial job demands | 14 | 37 | 113 | 279 | -^b^ | 200 | - | - | - | - |
| Work social support | 15 | 57 | 151 | 381 | - | 250 | - | - | - | - |
| Skill discretion | 11 | 42 | 116 | 287 | - | 209 | - | - | - | - |
| Decision authority | 19 | 49 | 115 | 302 | - | 209 | - | - | - | - |
| Sex | 0 | 0 | 0 | 0 | 0 | 0 | 0 | 0 | 0 | 0 |
| Partnership status | 12 | 3 | 3 | 150 | 66 | 10 | 1 | 68 | 28 | 18 |
| Occupational grade level | 0 | 7 | 4 | 21 | 75 | 17 | 332 | 234 | 0 | - |
| Left civil service | 0 | 0 | 0 | 4 | 0 | 1 | 2 | 9 | 3 | 23 |
| Depression medication intake | - | - | - | 4 | 4 | 2 | 0 | 1 | 0 | 0 |
| Chronic illness | 0 | 0 | 0 | 0 | 0 | 0 | 0 | 0 | - | - |
| Smoking status | 23 | 3 | 179 | 19 | - | 14 | - | 155 | 147 | 130 |
| Alcohol dependency | - | - | 18 | 97 | - | 82 | 103 | 274 | 166 | - |
| Body mass index | 5 | - | 167 | 908 | - | 261 | - | 296 | 292 | 238 |

Note: ^a^ Individuals are observed on average 8.2 times over phases 1-12. ^b^ ‘-’ indicates that variables were not measured in the respective phase, as described in Methods section of the main manuscript.
